# Specialized structures on the border between rhizocephalan parasites and their host’s nervous system reveal potential sites for host-parasite interactions

**DOI:** 10.1038/s41598-020-58175-4

**Published:** 2020-01-24

**Authors:** A. Miroliubov, I. Borisenko, M. Nesterenko, A. Lianguzova, S. Ilyutkin, N. Lapshin, A. Dobrovolskij

**Affiliations:** 1 Zoological Institute RAS, Laboratory of parasitic worms and protists, Universitetskaya emb., 1, Saint-Petersburg, 199034 Russia; 2 Saint-Petersburg State University, Department of Embryology, Universitetskaya Emb., 7/9, Saint Petersburg, 199034 Russia; 3 Saint-Petersburg State University, Department of Invertebrate Zoology, Universitetskaya Emb., 7/9, Saint Petersburg, 199034 Russia

**Keywords:** Fluorescence imaging, Optical imaging, Neurotrophic factors, Electron microscopy, Animal physiology

## Abstract

Rhizocephalan barnacles are a unique group of endoparasitic crustaceans. In their extreme adaptation to endoparasitism, rhizocephalan adults have lost almost all features of their free-living relatives but acquired an outstanding degree of control over the body of their hosts (mostly decapods). The subtle influence exercised by rhizocephalans on the physiology, morphology and behaviour of their hosts is a vivid example of the most intimate host-parasite interactions but their mechanisms are very poorly known. In this study we examined the morphology and the adaptive ultrastructure of the organs invading the nervous system of the host in two rhizocephalan species from the families Peltogastridae, (*Peltogaster paguri*) and Peltogasterellidae (*Peltogasterella gracilis*). We found two essentially different types of structures involved in interactions of these two rhizocephalans with the nervous system of their hosts: modified rhizocephalan rootlets lying inside the ganglia and the neural fibres of the host enlacing the trophic rootlets of the parasites. We suggest that both these structures may be highly specialized tools allowing the parasite to interact with the host on the humoral level via neuromediators, hormones, attractants and trophic factors.

## Introduction

Rhizocephala, or rhizocephalan barnacles, are a group of parasitic crustaceans that have lost almost all morphological characteristics of their free-living relatives due to adaptation to an endoparasitic lifestyle. These animals have a modified life cycle^1–4^ and a unique ontogeny resulting in a heavily transformed adult living as an endoparasite within other crustaceans, mostly decapods. In the life cycle of rhizocephalans, the adult organism originates from a group of poorly differentiated cells (“vermigon”) injected into the host by the larvae. Thus, the adult body of a rhizocephalan is newly formed and does not inherit any larval organs. It consists of two functional parts: an interna, which is a system of ramifying feeding rootlets spanning the body of the host, and an externa, which is a structure containing the reproductive system^5–9^.

All parasites influence their hosts at different levels (community, population, physiology of individual host) but the degree of influence varies. Here we will focus on the effect that a peculiar type of parasite (rhizocephalan barnacle) has on its host. Most parasites oppress their hosts by deriving nutrients from them but do not manipulate them. At the same time, some highly specialized parasites are known to modify physiology, morphology and behaviour of their hosts. This is characteristic of some trematodes, cestodes, nematodes and acanthocephalans^10–19^. Some metacercaria of trematodes are able to change the behavior of infected fish by modifying the concentration of monoamines in the brain^20^. Larvae of acanthocephalans induce serotonergic imbalance in a nervous system of their crustacean intermediate hosts^21^. Nematomorph hairworms alter the normal functions of their host’s (grasshopper) nervous system by the excretion of some special signal molecules^22^. However, not all taxa of parasites are well studied in terms of host-parasite manipulation. There are very few data about rhizocephalans, which are quite exceptional in their ability to take control over the host’s body.

Rhizocephalan barnacles show the highest level of integration with their hosts among metazoan parasites^23^ and the closest host-parasite interactions. They influence the moulting cycle, general physiology, morphology and behaviour of their hosts^6,23–30^. A vivid example of this influence is the feminization of the infested males^6,23,29,31^. Rhizocephalans are considered as parasitic castrators suppressing the reproductive system of the host^6,32^ but in contrast to some other parasitic castrators such as trematodes, which destroy the gonads of the host, rhizocephalan barnacles suppress gametogenesis. In addition, some of them take control over the moulting cycle and suppress moulting. This is important for their survival because moulting could result in damage or loss of the rhizocephalan externa, an organ of sexual reproduction positioned outside the host’s body^6,23,25–28,33^.

Infested male crabs demonstrate behaviour typical of females releasing larvae. However, the parasite forces the host to disseminate its own nauplii. The infestation by rhizocephalans reduces the level of aggressiveness and general activity of the infected crabs which might be considered a sort of manipulation also aimed at protecting the externa from damage^34–38^. At the same time, the voracity of the infected individuals increases^39^.

The mechanisms of control that rhizocephalans exercise over their hosts are still enigmatic. They may be expected to be highly species-specific and diverse. There is evidence that rootlets of the interna penetrate the neural ganglia within the ventral nervous cord of the host^40–42^. This is consistent with the significant metabolic and behavioural changes that infected crabs start to exhibit involving hormonal balance, respiration rate, hiding behavior, food consumption rate, aggressiveness and many others^34–39^. One may conjecture that there should be significant differences in histological structure and ultrastructure of the specialized rootlets invading the neural tissue and the trophic rootlets located within the body cavity of the host^6,26,27,29,40–42^. However, these differences have never been the focus of investigation.

In this study, we examined the morphology and adaptive ultrastructure of the organs invading the nervous system of the host in two rhizocephalan species, *Peltogaster paguri* from the family Peltogastridae and *Peltogasterella gracilis* from new established family Peltogasterellidae^43^. The aim of this study was to make a first attempt at revealing the structural mechanisms of interactions between rhizocephalans and their hosts. This study is going to be followed by biochemical and molecular investigations. In a broader context, our study is a first step towards understanding the integrative role of host nervous system for the parasite.

## Results

**Figure 1:**
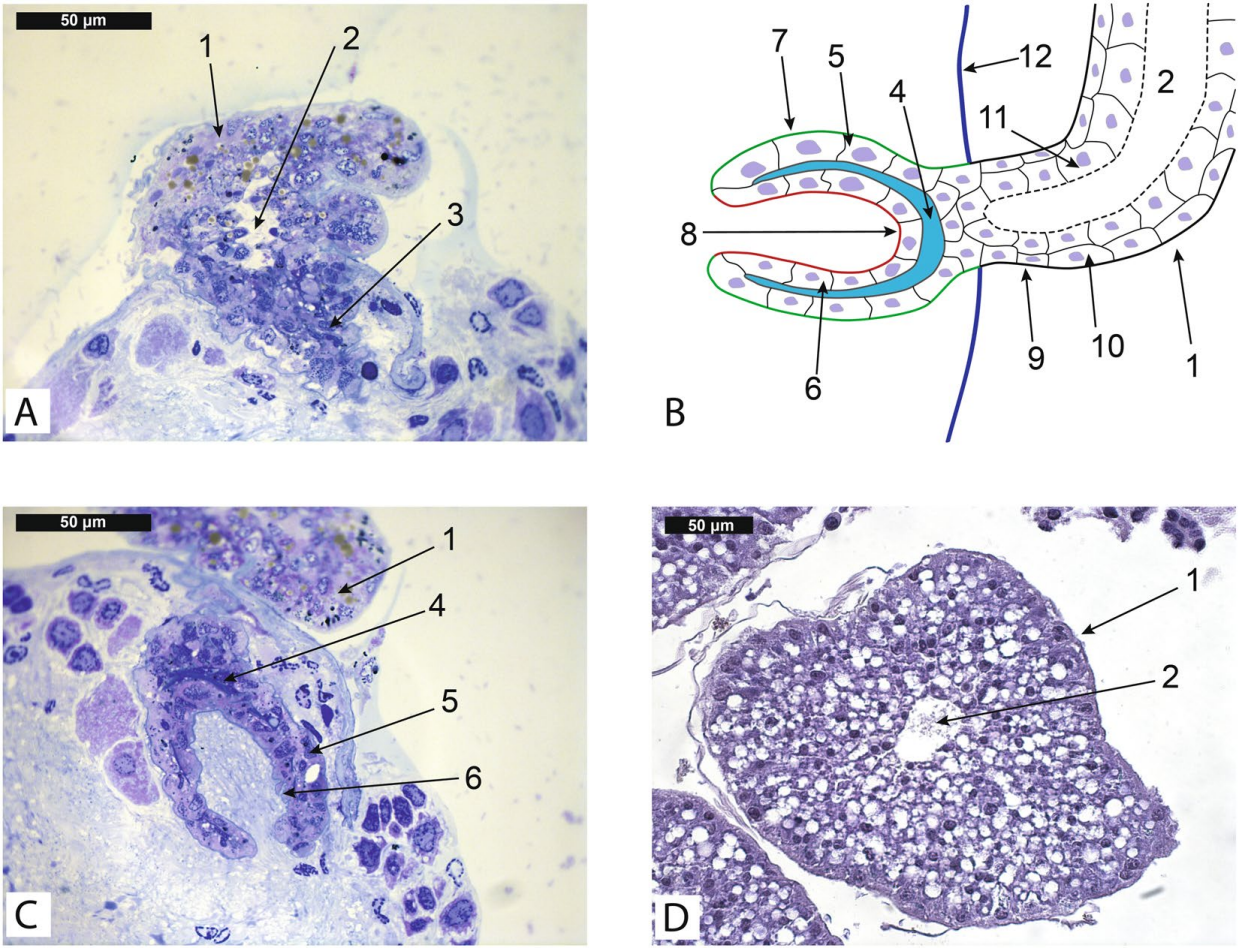
(**A,C**) – Histological sections of goblet-shaped organs (Peltogaster paguri) penetrating neural ganglion of the host (Pagurus pubescens). (**В**) – Scheme of goblet shaped organ of Peltogaster paguri. (**D**) – Histological cross section of the common trophic rootlet (Peltogaster paguri) in the hemocoel of the host (Pagurus pubescens). 1-rootlet associated with neural ganglion, 2-central lumen, 3-part of rootlet inside neural tissue, 4-extracellular matrix between layers of cells, 5-outer layer of cells, 6-inner layer of cells, 7-outer surface of goblet shaped organ (green), 8-inner surface of goblet shaped organ (red), 9-common cuticle on the surface of a rootlet, 10-layer of epithelial cells, 11-layer of axial cells, 12-ganglion envelope.

**Figure 2:**
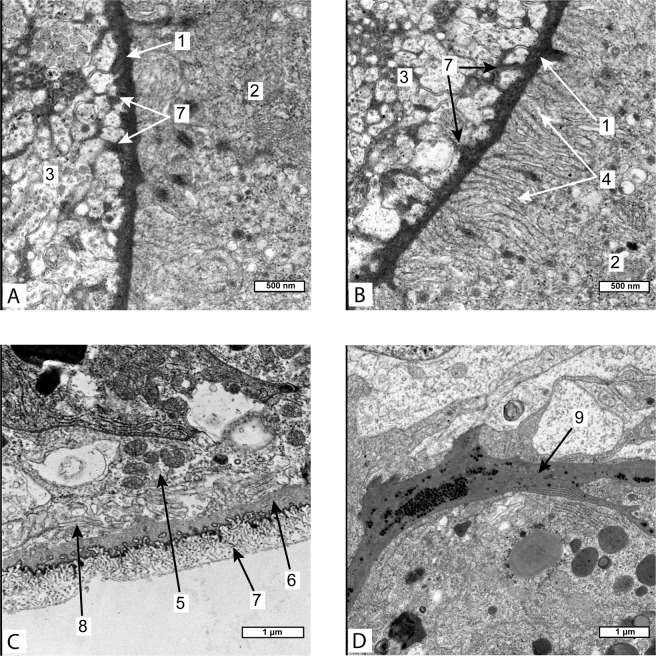
(**A,B**) – Ultrastructure of the cuticle on the internal surface of goblet-shaped organ of Peltogaster paguri, (**C**) – Ultrastructure of the cuticle of common trophic rootlet of Peltogaster paguri, (**D**) – Extracellular matrix between two layers of cells in goblet-shaped organ. (TEM) 1-cuticle on the internal surface of goblet-shaped organ, 2-cell of the parasite, 3-neural tissue of the host, 4-microville beneath the inner cuticle of goblet-shaped organ, 5-epithelial cell, 6-homogenous layer of cuticle, 7-microprojections of electron-dense layer of cuticle, 8-microville of epithelial cells, 9- extracellular matrix between two layers of cells in goblet-shaped organ.

Sections revealed that some roots of the interna of *Peltogater paguri* (Peltogastridae fam.) were associated with the first and the second abdominal neural ganglia of the host. It could be seen at histological sections that these roots penetrated the ganglion’s envelope (Fig. 1A,B) and that their distal parts were located in the mass of the neural tissue. The distal parts of these roots were modified into goblet-shaped structures with the funnel opening at the tip (Fig. 1C), which we will refer to as goblet-shaped organs. The average size of these organs in longitudinal direction was about 100 ± 10 μm. The tissue organization in goblet-shaped organs differed considerably from that in common trophic roots located in the body cavity of the host (Fig. 1D). The wall of goblet-shaped organs consisted of two layers of cells (an outer and an inner layer) and a layer of dark extracellular matrix containing dark round bodies (Figs. 1C, 2D).

The ultrastructure of the cuticle and the underlying cells in goblet-shaped organs also differed from that in trophic roots. The cuticle of a trophic root consisted of a delicate electron-dense outer layer and a thick homogeneous electron-translucent inner layer. The surface of the cuticle facing the host in most areas bore a zone of microprojections—extensions of the delicate electron-dense layer (Fig. 2C). The cuticle covering goblet-shaped organs differed on the inner and the outer surface of this organ. Inside the funnel (that is, on the inner surface of the goblet-shaped organ) it consisted of one electron dense layer with sparse microprojections (Fig. 2A,B) located between the neural fibres of the host. The apical surface of epithelial cells bore numerous microvilli arranged in a compact manner so the space between them was represented by small channels (Fig. 2B). In contrast, epithelial cells in the trophic roots formed apical microvilli with a wide subcuticular space (Fig. 2C). Whereas, the cuticle on the outer surface of the goblet-shaped organs lacked any microprojections, and there were no microvilli on the apical surface of the underlying cells. The cells of goblet-shaped organs contained numerous mitochondria and an abundant endoplasmic reticulum.

**Figure 3:**
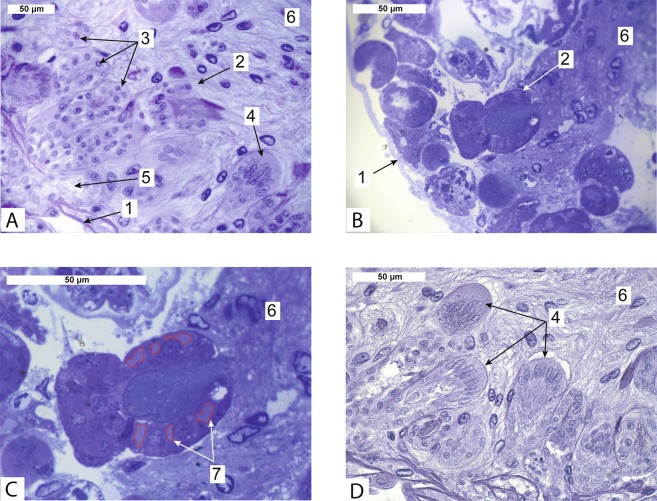
(**A–D**) – Rootlets of Peltogasterella gracilis inside neural ganglia of the host. (**A,D** – histological sections, **B,C** – semithin sections) 1-ganglion’s envelope, 2-goblet-shaped organ, 3-rootlets, 4-follicle like structure on the tip of growing rootlet, 5-rootlet penetrating the ganglion’s envelope, 6-neural tissue of the host, 7- vesicles with homogenous content inside cells of goblet-shaped organ (boarders of the vesicles pointed by the red line).

**Figure 4:**
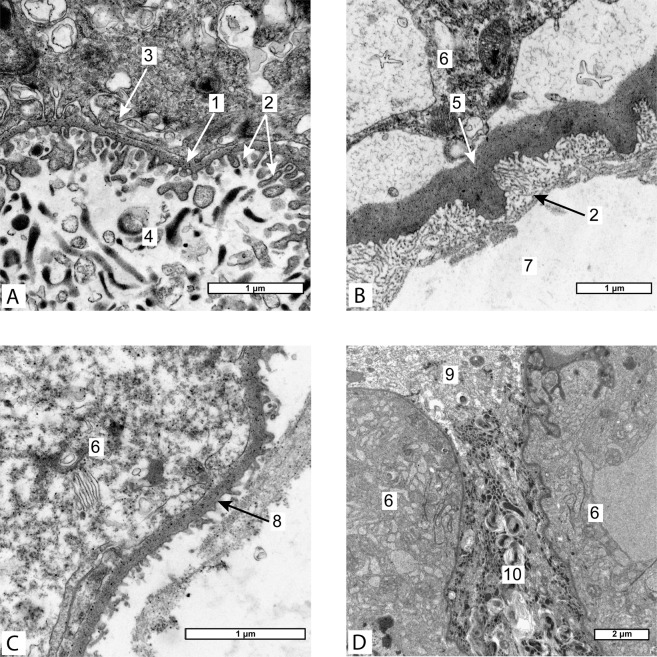
(**A**) – Ultrastructure of the cuticle on the internal surface of goblet-shaped organ of Peltogasterella gracilis, (**B**) – Ultrastructure of the cuticle of common trophic rootlet of Peltogasterella gracilis, (**C**) – Ultrastructure of the cuticle on the outer surface of goblet-shaped organ of Peltogasterella gracilis, (**D**) – longitudinal section through the opening of funnel of goblet-shaped organ of Peltogasterella gracilis. (TEM 1- cuticle, 2-cuticular micro projections, 3-microwilli beneath cuticle, 4 neural tissue of the host, 5- cuticle of common trophic rootlet, 6-cells of parasite, 7-body cavity of the host, 8- cuticle on the outer surface of goblet-shaped organ, 9-neural tissue of the host out of the goblet-shaped organ, 10-modified neural tissue inside goblet-shaped organ.

**Figure 5:**
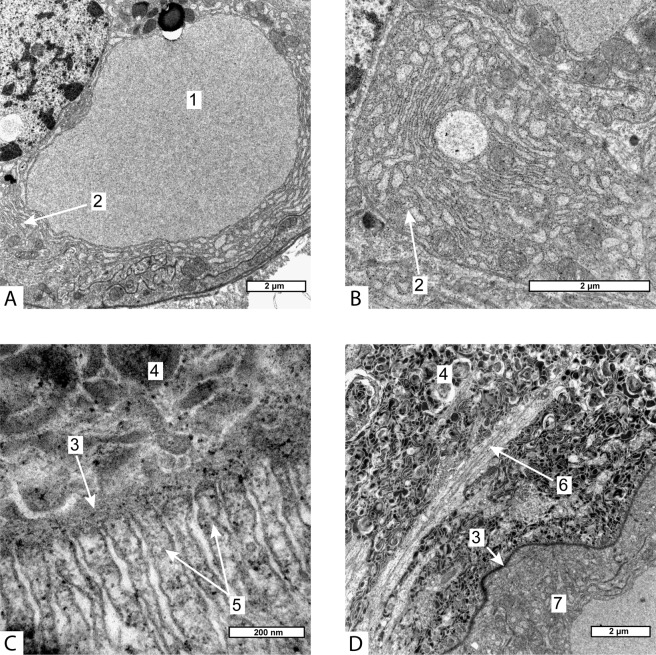
(**A,B**) – Bloated cisterns of endoplasmic reticulum in the cells of goblet-shaped organs of Peltogasterella gracilis, (**C**) – Ultrastructure of the cuticle on the internal surface of goblet-shaped organ of Peltogasterella gracilis, (**D**) – Ultrastructure of the modified neural tissue in the funnel of goblet-shaped organs of Peltogasterella gracilis. 1-bloated cisterns of endoplasmic reticulum, 2-endoplasmic reticulum, 3-cuticle on the internal surface of goblet-shaped organ, 4-neural tissue of the host, 5-microwille beneath the cuticle, 6-neural fiber of the host, 7-cells of goblet shaped organ.

Goblet-shaped organs of *Peltogasterella gracilis* (Peltogasterellidae fam.) had a similar gross morphology as those of *Peltogaster paguri* but differed somewhat in tissue organization and ultrastructure. In case of *Peltogasterella gracilis* goblet-shaped organs were much more abundant in the neural ganglia of the host than in case of *Peltogaster paguri* (Fig. 3A). Goblet-shaped organs of *Peltogasterella gracilis* were about twice smaller than those of *Peltogaster paguri*. Their average size in longitudinal direction was about 50 ± 10 μm. The wall of these organs in *Peltogasterella gracilis* consisted of a single layer of mainly polygonal cells (Fig. 3B,C). The cuticle of goblet-shaped organs in *Peltogasterella gracilis* consisted of two layers: a thin electron dense outer layer and a homogenous inner layer (Fig. 4A), which was similar to the cuticle structure in trophic roots (Fig. 4B). The surface of the cuticle inside the funnel of the goblet-shaped organs bore numerous projections lying between the elements of the host’s neural tissue but they were much thicker than the microprojections of trophic roots in *Peltogaster paguri*. Cells underlying the cuticle bore numerous long microvilli (Fig. 5C). On the outer surface of goblet-shaped organs, the cuticle also consisted of two layers and bore small sparse microprojections (Fig. 4C).

Large membrane bound vesicles with homogenous content were clearly seen in the cells of goblet-shaped organs of *Peltogasterella gracilis* (Fig. 3C), while the cells of *Peltogaster paguri* lacked any such structures. These vesicles were swollen cisterns of endoplasmic reticulum (Fig. 5A). We also observed empty vesicles located close to the endoplasmic reticulum (Fig. 5A), which were probably large vesicles with excreted content.

The effect on the host’s neural tissue was quite different in the two studied species. Despite the close contact with the parasite, the neural tissue of the host inside the funnel of goblet-shaped organs in *Peltogaster paguri* looked unmodified (Fig. 2A,B). Neural cells processes, synaptic structures, mitochondria and transport vesicles could be seen in cells located inside the funnel of the goblet-shaped organs. The fine structure of neural cells inside the funnel of goblet-shaped organs was indistinguishable of that of the cells outside. Inside the funnel of goblet-shaped organs in *Peltogasterella gracilis* the neural tissue of the host underwent changes resembling degeneration. The whole volume of the funnel of goblet-shaped organ was filled with some electron dense structures and membrane bodies similar to the structures characteristic of lysosomal autophagy, only few remnants of normal neural fibres could be rarely seen (Fig. 5D).

Besides goblet-shaped organs, roots with follicle-like structures on the tip were observed inside the host’s neural ganglia (Fig. 3D). These structures looked similar to the follicles on the tips of common trophic roots^23^. The wall of these structures consisted of a layer of columnar cells with oval nuclei as well as the common follicles on the tips of trophic roots. These follicles, observed in both species studied, seemed to be growing parts of the roots.

**Figure 6:**
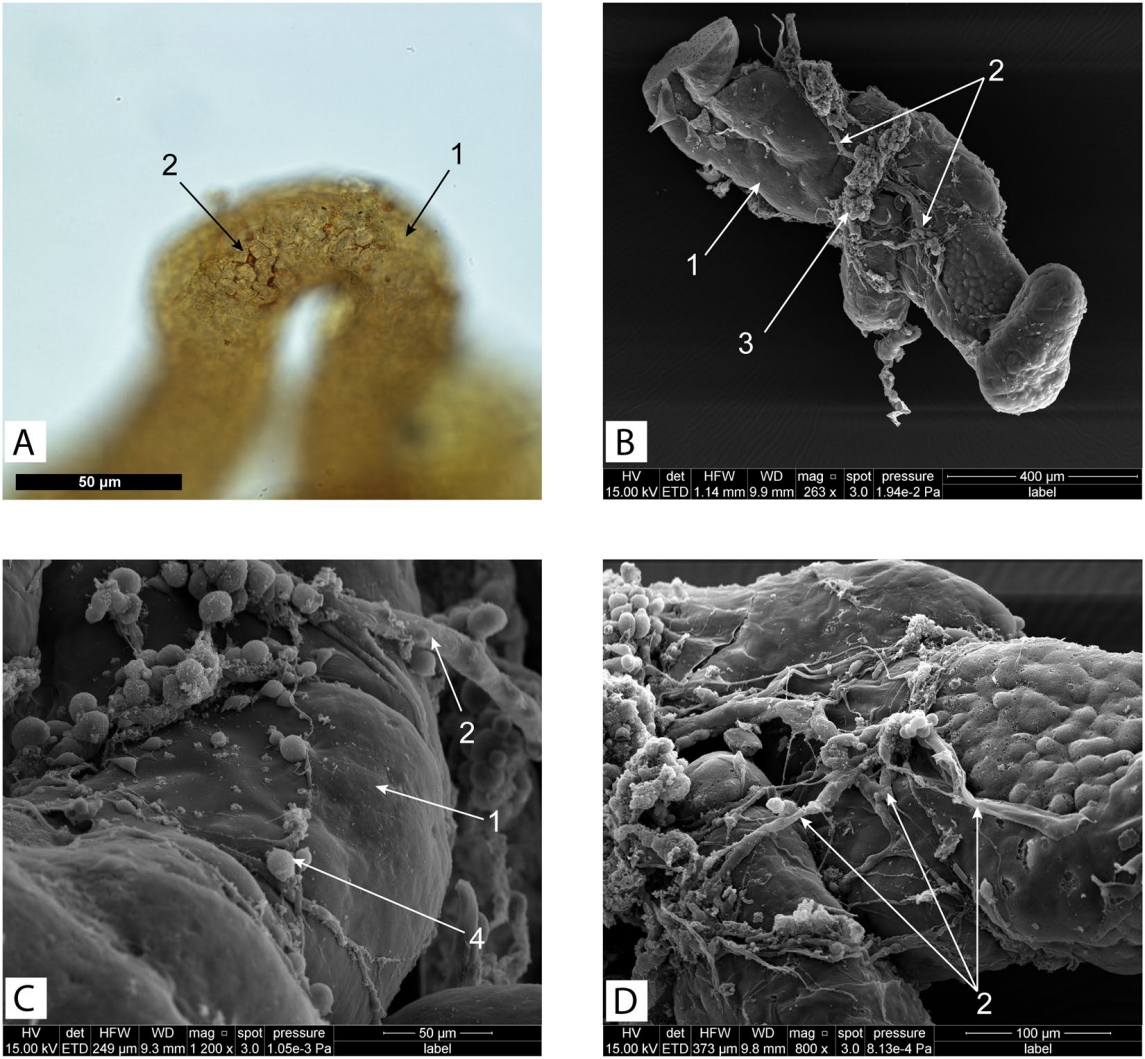
(**A**)- Cells stained with silver nitrate on the surface of the roots of *Polyascus polygenea*. (whole mount) (**B–D**)- host tissue on the surface of the roots of *Peltogaster paguri*. (SEM) 1-root, 2-fibers of host’s tissue, 3-groups of cells associated with fibers, 4-separate cells on the surface of the root.

**Figure 7:**
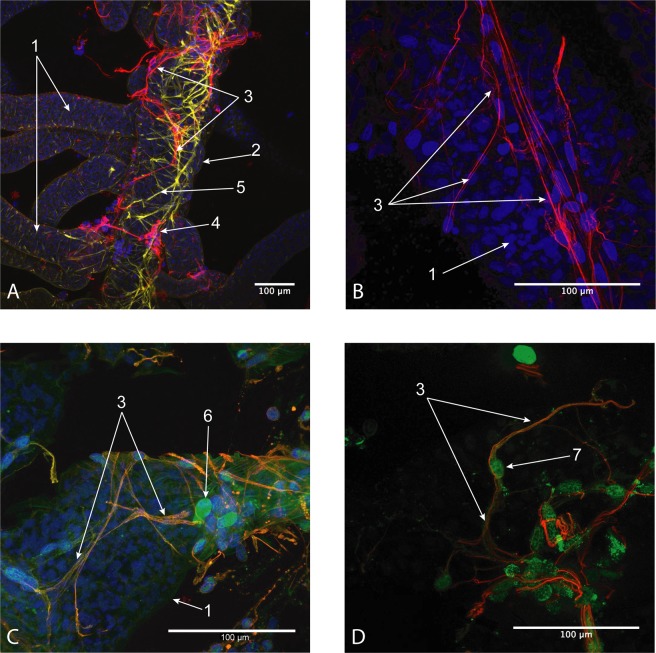
(**A,B**) – Main trunk and rootlet of *Peltogaster paguri* with neural fibers of the host (Confocal Z-stack), (**C**) – Neural fibers on the surface of rootlet of *Polyascus. Polygenea* (Sacculinidae fam.) (Confocal Z-stack), (**D**) – Neuron of the host on the surface of rootlet (*Peltogaster paguri*) (Confocal Z-stack). (Red**-**α-tubulin, Yellow-phalloidin, Blue-dapi, Green-Serotonin/FMRF-amid). 1-rootlets, 2-main trunk, 3-neural fibers of the host, 4-groups of cell bodies associated with neural fibers, 5-muscles, 6-body of neuron stained with FMRF-amide, 7- body of neuron stained with Serotonin.

We discovered that the trophic roots of *Peltogaster paguri* were enlaced by processes of the host cells. These processes, clearly seen as fibres on histological sections and SEM images (Fig. 6B–D), formed a network on the surface of trophic roots. The processes were α-tubulin-positive (Fig. 7A–D), while the cell bodies were serotonin- or FMRF-amid-positive (Fig. 7C,D), indicating the neuronal origin of these cellular structures. Host cells forming these processes resembled typical peripheral neurons in shape. In addition to antibody stainings and silver staining of rootlets of *Polyascus polygenea* revealed nervous network enlancing the rootlet (Fig. 6A).

## Discussion

In this study, we described two different types of structures involved in direct contact between rhizocephalans and the nervous system of their hosts: rhizocephalan rootlets modified into goblet-shaped organs and neural fibres of the host entwining the trophic internae. The presence of these structures might reflect the structural mechanism which facilitates interactions between rhizocephalans and their hosts.

In particular, we showed that ganglia-residing goblet-shaped organs in *Peltogaster paguri* and *Peltogasterella gracilis* differed from the common trophic roots in shape, ultrastructure and tissue organization. We hypothesize that they also have different functions. We did not find any synaptic contacts between the host tissue and the cells of goblet-shaped organs, which agrees with the fact that we never observed the nervous system inside parasitic interna^44^. This suggests that the interaction with the host nervous system may be mediated by soluble molecules produced by non-neural but nevertheless specialized tissue of the parasite. The ultrastructural features of the cells of goblet-shaped organs such as numerous microvilli beneath the cuticle, numerous mitochondria and abundant endoplasmic reticulum indicate high levels of local cellular synthesis and transport. We suggest that with the use of goblet-shaped organs, the parasite might inject specific substances such as neurotransmitters or neurohormones directly into the host’s neurons and could receive some other substances or neural-born signals from the host. That said, we point out that the permeability of the parasite’s cuticle for different molecules is unknown^26^.

The rest of the interna is located in the body cavity of the host and surrounded by the hemolymph and is considered to have a trophic function^6,26^. At the same time, the specific shape, tissue organisation and ultrastructure of goblet-shaped organs in comparison to the potentially trophic part of interna might indicate that they have a different set of functions. We suggest that goblet-shaped organs, which are located inside the neural tissue of the host and demonstrate an organization specialized for synthesis and transport, might also play a key role in modifying the physiological status, moulting cycle and behaviour of the host.

We observed specific differences in histology and ultrastructure of goblet-shaped organs in *Peltogaster paguri* and similar organs in *Peltogasterella gracilis*. The influence on the integrity of the host’s neural tissue also differed between these two species at the morphological level. *Peltogasterella gracilis* caused significant changes to the neural tissue of the host inside the funnel of goblet-shaped organs, whereas the neural tissue of the host of *Peltogaster paguri* seemed unmodified. The reason of these differences remains to be revealed. However, despite the differences in the ultrastructure and tissue organisation of the goblet-shaped organs in the two studied species, we hypothesize that they are functionally analogous because their gross morphology and localisation are quite similar. Morphological examination of the goblet-shaped organs of both species described above was repeated in a number of specimens and all the examined parasites were sexually mature so we can confirm that there were differences between these two species.

Goblet-shaped organs are located inside of the neural ganglia of the host but it is unknown how they germinate and penetrate the ganglion’s envelope. We speculate that the parasitic follicles observed inside the host’s ganglia (Fig. 3D) are, in fact, a stage of the developing goblet-shaped organs responsible for penetrating the ganglia and their connective tissue sheaths. These follicles might excrete factors diluting the ganglial envelope and develop into mature goblet-shaped organs after penetration.

Penetration of interna rootlets into the ganglia of ventral nervous cord was shown in several studies. According to Nielsen (1970)^40^, some roots of *Peltogaster paguri* and *Peltogasterella sulcata* were associated with the posterior part of thoracic ganglion and the next three abdominal ganglia. The rootlets penetrated the ganglial envelope and were located in the neural tissue. Structures similar to goblet-shaped organs can be clearly seen in the figures in that article (Nielsen, 1970, p. 26) but were not described there. Similar structures were also found inside the ganglia of crabs infested by sacculinid parasites^41^. Unfortunately, they were described only at the histological level. However, the fact that representatives of other families have similar organs points to the common trends of interactions between parasitic barnacles and the nervous system of their hosts.

The phenomenon of nervous fibres enlacing the trophic roots of the interna discovered in our study is intriguing and suggested many further questions. This is another site of direct contact of the parasite with the nervous system of the host and its role may be different from that of goblet shaped organ. We suggest that the parasite might induce the growth of neurons by emitting some analogues of the host’s neuronal attractants, which force neurons to grow on the surface of the roots and to enlace them. The purpose of this is unclear but, as a further conjecture, this site might serve for direct host-parasite interactions.

To conclude, rhizocephalan barnacles of these both species (*Peltogaster paguri* and *Peltogasterella gracilis*) have at least two distinct types of direct contact with the neural system of the host: goblet shaped organs and the neural fibres entwining the rootlets. Both of these structures may be highly specialized tools allowing the parasite to interact with the host. Molecular mechanisms for these interactions might be a promising direction of future studies. Interaction with the nervous system of the host is all the more fascinating as rhizocephalan interna has never been shown to have its own nervous system^44,45^. At the same time, its muscular system is well-developed^45^ but it is unclear how it is innervated. While neural tissue is absent in parasite body it is still unknown where muscular contraction originate and how they are transmitted.

## Materials and Methods

Hermit crabs *Pagurus pubescens* (Krøyer, 1838) (17 specimens) infected with the parasite *Peltogaster paguri* (Rathke, 1842) were collected at the White Sea (Educational and research station “Belomorskaia” of St Petersburg State University) (N: 66.308210, E: 33.627816) in the summer of 2016 and 2017. Hermit crabs *Pagurus ochotensis* (Brandt, 1851) and *Pagurus pectinatus* (Stimpson 1858) (15 specimens) infected with *Peltogasterella gracilis* (Boschma, 1927) and crabs *Hemigrapsus sanguineus* (De Haan 1835) infected with *Polyascus polygenea* (Lützen, J. & T. Takahashi 1997) (10 specimens) were collected at the Sea of Japan (Marine biological station “Vostok” of Institute of Marine Biology of the Russian Academy of Sciences) (N: 42.893720, E: 132.732755). All collected parasites were adults with fully developed externas. The infected hosts were dissected and the ventral nerve cords with the roots of rhizocephalan parasites were removed.

### Immunocytochemistry (ICC)

For immunocytochemical visualization the internae of the rhizocephalans were fixed with 4% paraformaldehyde (PFA; Sigma-Aldrich) in phosphate-buffered saline (PBS; Fluka) at 4 °C overnight. Before immunocytochemical staining, the fixed material was rinsed with PBT several times in the course of 24 hours (PBS + 0.1% Triton-X100; Sigma-Aldrich). The specimens were blocked with 1% bovine serum albumin in PBT for an hour and incubated with primary antibodies diluted in blocking solution overnight at 4 °C. The primary antibodies used were anti-tyrosine α-tubulin (mouse monoclonal, 1:2,000; Sigma Cat #T9028) and anti-acetylated α-tubulin (mouse monoclonal, 1:2,000; Sigma Cat #T6793). After incubation with primary antibodies, the samples were washed four times (for 3 hours each time) in PBT, and incubated for 12 hours at 4 °C with a 1:1000 dilution of Donkey Anti-Mouse IgG Antibodies labelled with Alexa Fluor 488 (Molecular Probes, Cat #A21202). The material was then rinsed three times for 10 min each time in PBS, stained with the DAPI nuclei stain (1 ug/ml; Sigma) in PBS for an hour, rinsed with PBS and mounted in DABCO-glycerol. The specimens were examined using the confocal laser scanning microscopes Leica TCS SPE in the Resource Centre “Microscopy and Microanalysis” of Research park of St State University. The images were processed using ImageJ software (FiJi).

### Histology and light microscopy

The dissected internae were fixed with Bouin solution. Paraffin sections (5 μm thick) were made using standard histological methods on the Leica RM-2265 microtome and stained with hematoxylin-eosin. The sections were examined under a Leica DM2500 microscope, photos were taken with a Nikon DS-Fi1 camera and processed with ImageJ software (FiJi).

Interna of *Polyascus polygenea* (fam. Polyascidae) isolated from the host was rinsed in fresh water and incubated in silver nitrate solution for 24 hours, after that whole specimen was mount on slides. This method is used to visualize the nervous system.

### Transmission and scanning electron microscopy

For transmission electron microscopy samples were fixed overnight at 4 °C in 2.5% glutaraldehyde in phosphate buffer with addition of Sucrose (pH 7.4; 750 mOsM), and postfixed in 1% OsO4 in the same buffer (an hour at room temperature). After washing with the same buffer, the specimens were dehydrated through an ethanol series and acetone and embedded in Epon-812 embedding media (Fluka). Semithin (1 μm thick) and ultra-thin (60–80 nm) sections were cut with a Leica EM UC7 ultratome. Semi-thin sections were stained with methylene blue and studied under Leica DM2500 microscope. Ultra-thin sections were stained with uranyl acetate followed by Reynolds lead nitrate^46^ and examined under transmission electron microscope JEOL JEM 1400 in Resource Centres “Chromas” and “Molecular and Cell Technologies” of the Research Park of St Petersburg State University. Specimens for SEM were dehydrated in ethanol series and acetone, critical point-dried in Hitachi critical point dryer HCP- 2, mounted on stubs, coated with platinum using Giko IB-5 Ion coater, and viewed under FEI Quanta 250 scanning electron microscope in “Taxon” Research Resource Center (http://www.ckp-rf.ru/ckp/3038/) of the Zoological Institute of the Russian Academy of Sciences.
